# Genomic Characterization of Novel *Listeria monocytogenes* Serotype 4b Variant Strains

**DOI:** 10.1371/journal.pone.0089024

**Published:** 2014-02-19

**Authors:** Pongpan Laksanalamai, Bixing Huang, Jonathan Sabo, Laurel S. Burall, Shaohua Zhao, John Bates, Atin R. Datta

**Affiliations:** 1 Center for Food Safety and Applied Nutrition, US Food and Drug Administration, Laurel, Maryland, United States of America; 2 Public Health Microbiology Laboratory, Queensland Health Forensic and Scientific Services, Queensland, Australia; 3 Center for Veterinary Medicine, US Food and Drug Administration, Laurel, Maryland, United States of America; Naval Research Laboratory, United States of America

## Abstract

Over 90% of the human listeriosis cases are caused by *Listeria monocytogenes* serotypes 1/2a, 1/2b and 4b strains. As an alternative to antigen-antibody based serotyping, a PCR-based method for serogrouping has been developed and validated. In this communication, we report an in-depth analysis of five 4b variant strains, four clinical isolates from Australia and one environmental isolate from USA. Although these five strains were serotype 4b by classical serotyping method, the serogrouping PCR profiles of these strains show the presence of a 1/2a-3a specific amplicon in addition to the standard 4b-4d-4e specific amplicons. These strains were further analyzed by pulsed field gel electrophoresis, binary gene typing, multi-locus variable-number-tandem-repeat analysis and a high density pan-genomic *Listeria* microarray. Using these sub-typing results, the clinical isolates were grouped into two distinct genomic groups- one of which could be part of an unidentified outbreak. The microarray results when compared with our database of other 4b outbreak isolates indicated that the serotype 4b variant strains represent very different genotypic profiles than the known reported 4b outbreak strains representing major epidemic clones. The acquisition of serotype 1/2a gene clusters by the 4b variant strains appears to be independent in origin, spanning large areas of geographical and temporal space and may indicate predisposition of some 4b strains towards accepting DNA from related organisms.

## Introduction

*Listeria monocytogenes* continues to cause foodborne diseases with 20–30% mortality and >95% hospitalization. The incidence of foodborne listeriosis in the United States alone is about 1,600 cases per year [1]. The incidence in most of the European countries and Canada is similar with slightly higher rates in Scandinavian countries [2]. Although the majority of listeriosis outbreaks and sporadic cases have been associated with deli meats and dairy products, recent listeriosis outbreaks involving fresh fruits and vegetables, including the cantaloupe associated outbreak in the US, are indicative of the fact that *L. monocytogenes* can survive and multiply in foods other than those commonly reported as a vehicle for foodborne listeriosis [3], [4]. Also interesting is the noticeable shift in demography of the individuals contracting listeriosis. During 1980–2000, most of the listeriosis cases were pregnancy associated while recent outbreaks show that the majority of the cases were non-pregnancy associated affecting elderly individuals [5], [6]. These observations underline the importance of in-depth genomic characterization and their significance in understanding the emergence of newer pathotypes, association with newer food groups and the shift in demography.

The value of molecular sub-typing for *Lisetria* and other foodborne pathogens during outbreak and traceback investigations cannot be overemphasized. In addition to epidemiological investigation, accurate determination of the source/s of foodborne outbreaks by comparing molecular sub-typing patterns of clinical, food and environmental isolates provides the scientific basis for quick determination of contaminated food/s thereby reducing the spread and burden of the outbreaks. In addition, molecular sub-typing is also important for understanding the pathophysiology [7], [8] of the organisms, source attribution [9] and for understanding of genomic evolution and emergence of newer traits. For example, that there may be specific genetic footprints in strains causing febrile gastroenteritis and invasive listeriosis was evident from the DNA microarray based sub-typing of *L. monocytogenes* [7] but the significance of such findings is far from clear. Previous studies have also indicated that different sub-types of *L. monocytogenes* are not equally distributed among food, environmental and clinical samples and different sub-types may pose different amount of risks [10], [11]. In order to identify the genetic diversity among the outbreak strains, several molecular approaches have been utilized [12]. These efforts clearly show the usefulness of detailed genotypic characterization of the outbreak associated *L. monocytogenes* strains not only for epidemiological and trace-back investigations but also for understanding the diversity and evolution of this organism. It is anticipated that in-depth genomic characterization of *L. monocytogenes* strains will help formulate intelligent hypotheses for its diverse pathophysiology, adaptation to newer food matrices and change in disease demography.

Serotyping of *L. monocytogenes* constitutes the very first step of sub-typing. Based on somatic and flagellar antigens, *L. monocytogenes* can be classified into 13 serotypes [13] of which serotypes 1/2a, 1/2b and 4b represent the vast majority of the disease causing strains [14]–[16]. The classical serotyping based on antigen-antibody reaction is time consuming, complicated and subjective [15], [17]. A simpler version of the classical serotyping is the determination of serogroups 1 and 4 by slide agglutination assay. The test, although simple, does not identify serotypes nor does it identify serogroup 3. Additionally, the test is variable and subject to interpretation. To avoid the usual pitfalls of the antigen-antibody based serotyping, several genome sequence-based serotyping methods have been developed [18]. Of all these sequence-based methods, a simple multiplex PCR based method by Doumith et al (2004) [19] appears to hold the maximum promise. The PCR-based assay uses five primer pairs of which four of them are serogroup specific and the fifth one, primers for *prs* gene, is *Listeria* genus specific [17], [19]. A modified version of this assay using *hly* specific primers instead of *prs* gene specific primers for *L. monocytogenes* has been reported by Burall et al [17]. Using this scheme, while majority of the strains can be properly classified in 1/2a-3a, 1/2b-3b, 1/2c-3c and 4b, 4d, 4e groups, a small group of strains showed PCR banding patterns that could not be classified by this scheme. These 4b variant strains, termed IVb-v1, produced a serotype 1/2a specific lmo0737 amplicon in addition to standard serotype 4b,4d, 4e specific bands for ORF 2110 and ORF 2819 [17], [20]. Recently, Leclercq et al (2011) [21] and Lee et al (2012) [22] reported the characterization of 45 serotype 4b *L. monocytogenes* strains collected over a long period from the different parts of the world. These IVb-v1 strains have been isolated from a variety of food, human and environmental sources separated by time and space. The multiplex serotyping PCR [19] of these strains also resulted in an *lmo*0737 specific band in addition to serogroup 4b specific bands. LeClercq et al. analyzed 22 IVb-v1 strains by *Apa*I/*Asc*I generated PFGE profiles and grouped these strains into six different profiles, although 14 out of 22 strains were indistinguishable from each other indicating that there are clonal groups among these IVb-v1 strains. A sub-set (n = 7) of these strains were also analyzed by a multi locus sequence typing (MLST) protocol which revealed two MLST types [21]. The twenty three strains from clinical and processing plants from US was similarly analyzed by Lee et al. [22] by MLGT and susceptibility to Sau3A/MboI digestion and found to form three clonal groups.

In this paper, we report an in-depth genetic analysis of a group of five IVb-v1 strains originated in two different continents, Australia and North America, by a variety of sub-typing methods including pulsed field gel electrophoresis, binary typing, multi-locus variable tandem repeat, restriction enzyme digestion and a custom made pan-genomic DNA microarray. These techniques with varied discriminatory indices [23] provided us with a unique opportunity to compare the usefulness of multiple sub-typing techniques for their use during outbreak investigation and other purposes. Our results showed that the three of the four IVb-v1 strains from Australia probably represent an undocumented outbreak cluster. These three IVb-v1 strains also appear to form a separate clonal group, distinct from other clonal groups reported for the IVb-v1 strains [22]. Results from these molecular sub-typing assays identified unique genetic footprints of these strains and discuss the value of such analyses to understand genomic diversity, evolution and biology of *L. monocytogenes.*

## Materials and Methods

### Serotyping by Antisera and by PCR

The *L. monocytogenes* serotype 4b variant strains (Table 1) were serotyped by multiplex PCR and antisera agglutination as described previously [17]. Briefly, overnight cultures grown on BHI agar at 37°C were used to make lysates for multiplex PCR analysis as well as for agglutination assay using Difco *Listeria* types 1 and 4 antisera (BD Diagnostic Systems, Sparks, MD) following the manufacturer’s protocol. A commercially available *L. monocytogenes* serotyping kit (Denka Seiken Co., Tokyo, Japan) was also used to serotype some of these isolates using the manufacturer’s instruction.

**Table 1:** *L. monocytogenes* strains used in this study and their serotypic profiles.

Strains	Source/Symptom	Multiplex PCR						Serotype**
		ORF 2819	ORF 2110	*Lmo* 0737	*Lmo* 1118	*prs*	*hly*A	
LS406	Human/Febrile Gastroenteritis	+	+	−	−	+	+	4b
LS412	Human/Invasive	+	+	−	−	+	+	4b
LS542	Environmental Swab	+	+	+	−	+	+	4b
LS642(10M127*)	Human/Invasive	+	+	+	−	+	+	4b
LS643(10M130*)	Human/Invasive	+	+	+	−	+	+	4b
LS644(10M138A*)	Human/Invasive	+	+	+	−	+	+	4b
LS645(10M198*)	Human/Invasive	+	+	+	−	+	+	4b

*Alternate designation [20].

**Antibody- based serotyping.

+/− indicates presence/absence of the band.

### Binary Typing

A binary typing method was developed based on the presence or absence of selected genes among the *L. monocytogenes* isolates [24]. Out of 44 screened candidate genes, an eight loci panel showed more significant variations than others. This eight loci panel combined with PCR-based serotyping [19] provided 95.4% Simpson index (SI) as a typing tool [23].

### Multilocus Variable Tandem Repeats Analysis (MLVA)

Multi-locus variable-number-tandem-repeat analysis (MLVA) is a widely used typing method for *L. monocytogenes* [25]–[28]. An optimized MLVA typing panel was developed recently by selection of the optimal combination of loci from the previously reported panels [29]. Therefore, we used this new method for typing of the 4b variant strains.

### Pulsed-field Gel Electrophoresis (PFGE) Typing

Pulsed-field gel electrophoresis (PFGE) analysis was performed according to the protocol developed by the Centers for Disease Control and Prevention (CDC, Atlanta, GA; http://www.cdc.gov/pulsenet/protocols.htm), using *Salmonella braenderup* H9812 as the control strain. PFGE results were analyzed using the BioNumerics Software (Applied-Maths, Kortrijk, Belgium). Banding pattern similarity was compared using an average of two-enzyme analysis with a 1.5% band position tolerance. All PFGE profiles generated were compared to isolates from clinical human Listeriosis cases in the CDC national PulseNet database.

### DNA Microarray Analysis

The *L. monocytogenes* serotype 4b variant strains (Table 1) were grown in brain heart infusion (BHI) broth and/or BHI agar at 37°C. The *L. monocytogenes* 4b strains used in the comparison of genomic contents were obtained from various sources previously described [7]. Genomic DNA was isolated from 10 ml of cultures grown overnight in a shaking incubator at 170 rpm using the Qiagen DNeasy Blood and Tissue kit (Qiagen, Valencia, CA), followed by DNA fragmentation and 3′-end labeling as previously described [7]. The labeled product was then used for hybridization onto the *Listeria* GeneChip. Array hybridization, washing, staining and scanning were performed from the labeled DNA according to the Affymetrix GeneChip Expression Analysis Technical Manual and Laksanalamai et al. 2012 [7], [30].

All Affymetrix CEL files generated in this study were parsed and analyzed using algorithms including MAS5.0 [30]–[32] with a *Tau* value as reported previously [7]. Robust Multi Array (RMA) method to identify summarized probe-set intensities was implemented by the Affy package of R and Bioconductor [33]–[36]. The gene present/absent binary nucleotide calls were performed as described previously [7] and the genetic relationship among these strains were analyzed using Splitstree 4.11.3 [37]. A neighbor-net or neighbor joining phylogeny highlighting the distribution of the *L. monocytogenes* serotype 4b variant strains was constructed using the uncorrected *p*-distance in Splitstree 4.11.3.

### Restriction Enzyme Digestion Analysis

Genomic DNA, prepared as described previously, was used for restriction digest analysis with MboI and Sau3A1 (New England Biolabs, Ipswich, MA) according to the manufacturers protocols, similar to prior work [38]. Reactions, containing 0.5 ug of genomic DNA, were incubated for 1hr at 37°C and then loaded on a 1% agarose gel for visualization of DNA digestion.

## Results and Discussion

**Figure 1:**
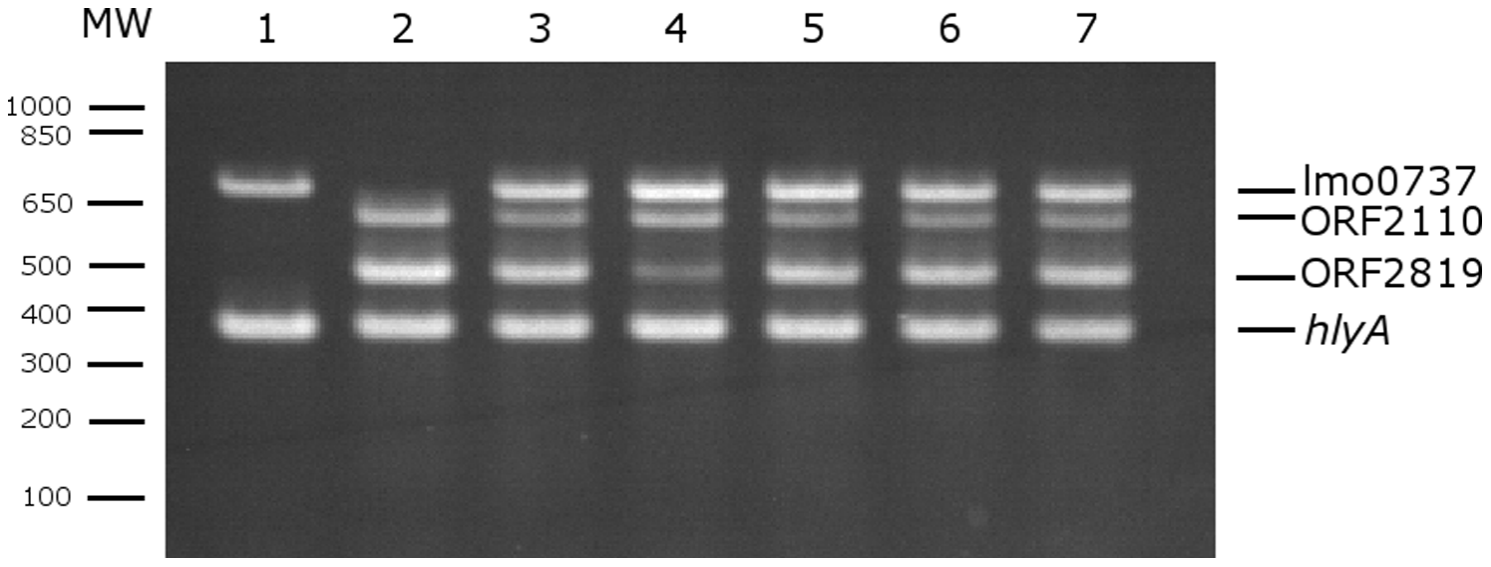
Multiplex PCR profiles of *L. monocytogenes* strains. Lanes: MW, Molecular weight markers. Lane 1: LS1 serotype 1/2a, Lane 2: LS402, serotype 4b; Lane3-7: serotype IVb-v1 strains LS542; LS642; LS643; LS644; LS645, respectively.

The three clinical isolates, LS642, LS643 and LS644, were from listeriosis patients in New South Wales, Australia and the fourth clinical isolate, LS645, was from Victoria, Australia in 2009. The isolates were collected as a part of routine surveillance of human listeriosis cases in Australia and no epidemiological link was identified among these four cases [20]. The fifth isolate, LS542, was from a soft cheese manufacturing facility environment, collected as a part of monitoring of ready to eat food facilities by USFDA [17]. The isolates were serotyped by antisera serotyping kit as 4b. However, the results of the multiplex PCR serotyping appeared to be a combination of serogroup 4b-4d-4e and 1/2a-3a (Fig. 1). These strains produced three serogroup specific bands of *lmo0737*, ORF2110 and ORF2819 in addition to *Listeria* genus specific *prs* band and *L. monocytogenes* specific *hly* band confirming that the isolates were indeed *L. monocytogenes*. The additional PCR band, *lmo*0737 is characteristic of serogroup 1/2a-3a. The amplified band for *lmo*0737 from these five strains were purified and sequenced and the sequence comparison between these fragments and bona fide *lmo*0737 fragment from a 1/2a strain showed that the sequences of all six strains were identical (data not shown) establishing that the additional band was indeed from *lmo*0737 and not from any other sequences resulting from any mis-priming or other artifacts during the amplification. In order to further characterize these five IVb-v1 strains, we conducted binary gene typing [24] of these strains. Table 2 shows the results from this assay. LS643, LS644 and LS645, clinical isolates from NSW and Victoria formed a single binary type 158 while LS642, the single clinical isolate from NSW and the environmental isolate from the USA, formed their own types; LS542 appeared to be genetically closer to LS642 as they differed by a single locus LC32. Similar genotypic variability was also observed by MLVA (Table 3). The MLVA analysis [29] grouped LS643, LS644 and LS645 into a single pattern (04-17-24-05-02-0-15-0-21) while LS542 and LS642 varied in five out of nine loci analyzed in this assay. These results clearly indicate that LS643, LS644 and LS645 represent a clonal group. Although no epidemiological link could be established, the results strongly indicate the possibility of a common source for these cases.

**Table 2:** The binary gene typing profiles of the *L. monocytogenes* strains.

Strains				Genetic	Loci				Binary Type
	LC3	LN4	LB10	LC32	LB50	LC52	LC68	LN1	
LS542	+	−	+	−	+	+	+	+	175
LS642	+	−	+	+	+	+	+	+	191
LS643	+	−	−	+	+	+	+	−	158
LS644	+	−	−	+	+	+	+	−	158
LS645	+	−	−	+	+	+	+	−	158

+/− indicates presence/absence of band.

**Table 3:** The MLVA profiles of the *L. monocytogenes* strains.

Strains				Genetic	Loci				
	LMV6	LMV1	LMV2	Lm11	Lm10	LMV7	Lm32	LMTR6	Lm23
LS542	05	06	23	05	02	07	14	0	14
LS642	03	12	14	05	03	07	14	0	16
LS643	04	17	24	05	02	0	15	0	21
LS644	04	17	24	05	02	0	15	0	21
LS645	04	17	24	05	02	0	15	0	21

Numbers in the boxes represent the numbers of repeats in each of these loci.

**Figure 2:**
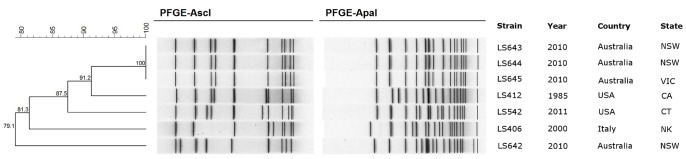
*ApaI/AscI* PFGE profiles of *L. monocytogenes* strains 4b and IVb-v1. The dendrogram was calculated and drawn using Bionumerics software. NSW; New South Wales, VIC; Victoria, CA; California, CT; Connecticut, and NK;Not known.

**Figure 3:**
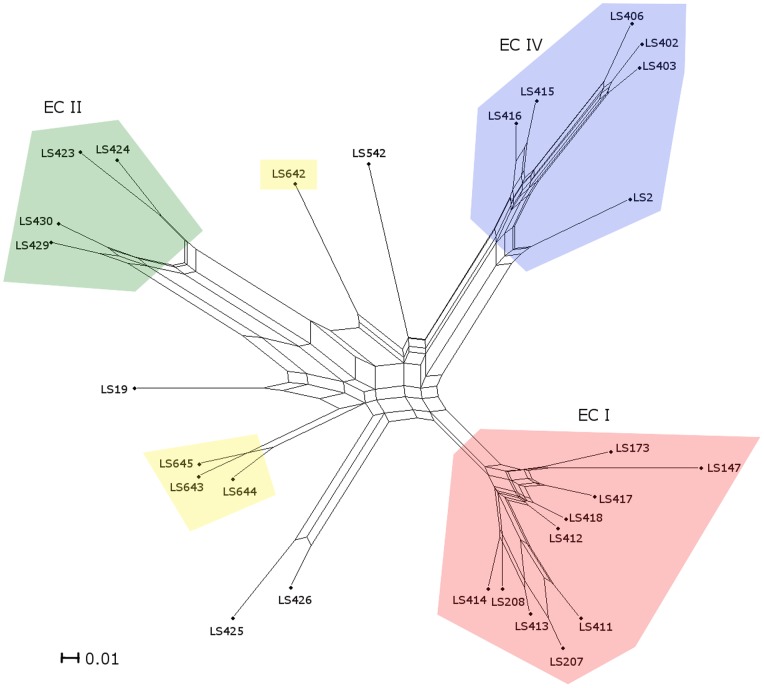
A neighbor-net constructed from the gene contents of 28 strains belonging to serotype 4b. The parallel edges represent incompatible signals indicative of independent gene loss or gain due to the multiple transduction or recombination. Serotypes and epidemic clones are grouped in different color as indicated. Node labels refer to strain names (Table 1 and Laksanalamai et al 2012 (7). Scale bar represents number of gene differences (present or absent) per gene site.

The PulseNet database stores and analyzes PFGE profiles of various foodborne bacterial pathogens including *L. monocytogenes* [39]. Since its inception, PulseNet has become the mainstay in foodborne outbreak investigations in the USA and rest of the world. Currently, the US PulseNet *L. monocytogenes* database contains more than 13,000 PFGE patterns, including isolates from human (n = 7576), animal (n = 46), food (n = 2863) and environment (n = 2799). In order to further characterize the genotypic variability of these IVb-v1 strains, we performed PFGE analysis of these strains using a standard PFGE protocol [40]. Figure 2 shows the graphical representation of the *ApaI/AscI* PFGE profiles of five IVb-v1 and two serotype 4b strains (Table 1) representing an invasive outbreak LS412 and a gastroenteritis outbreak LS406. The PFGE profiles were analyzed and the dendrogram was drawn as described in the Materials and Methods. Again, it is clear that the PFGE profiles of LS643, LS644 and LS645 were indistinguishable from each other but they are quite distinct from LS542 and LS642 (Fig. 3). A query of the PulseNet database of the *L. monocytogenes* PFGE profiles did not reveal any match with any of these three patterns arising from the IVb-v1 strains although a few closely matched patterns with LS542 have been observed (data not shown). Overall, the PFGE data mirrored the binary typing and MLVA data indicating that LS643, LS644 and LS645 are genotypically very similar to each other and may indicate a common source for all three human cases. Although we did not see any significant difference among these three typing methods, the PFGE based typing provided an opportunity to compare these strains with other strains collected over the years and stored in the PulseNet dataset.

High density DNA microarray has been successfully utilized for species identification [41], [42], serotypic and lineage determination [43], [44], virulence assessment [45] and epidemiological investigations [46], [47]. A pan-genomic microarray, *Listeria* GeneChip, has been used to reveal the genomic contents of *L. monocytogenes* outbreak strains [7] including strains involved in the 2011 cantaloupe associated listeriosis outbreak [4]. In this study, we also used the same *Listeria* GeneChip to analyze the gene contents and genomic architecture of the IVb-v1 strains.

The comparison of the probe-set data (present/absent) between the serotypes 4b and IVb-v1 by MAS5.0 algorithm revealed that strains LS643, LS644 and LS645 are clustered together forming a new group that is distinct from other serotype 4b outbreak strains. The genetic content analysis of strain LS642 clearly indicated that it is branched away from these three strains (Fig. 3) suggesting extensive genetic variability. Although the strain LS642 was isolated from the same state of New South Wales, it is more closely related to the strain LS542 than to its Australian counterparts. The microarray results combined with the PFGE, binary genotyping and MLVA data again support the notion the strains LS643, LS644, and LS645 could be part of a common source outbreak even though no epidemiological link was established. The microarray data also allowed us to identify if these strains belonged to any of the known epidemic clones (EC). Previously, extensive genomic information has led to the establishment of five distinct ECs of *L. monocytogenes* [7], [14] of which serotype 4b strains belonged to ECI, ECII and ECIV. Our microarray data clearly shows that the IVb-v1 strains are genotypically distinct and do not belong to any of these three ECs (Fig. 3). Comparison of the gene contents between LS642 and the group of LS643, LS644 and LS645 revealed that 2.2% (415) of all the probe-sets are uniquely present in strain LS642 but absent in all of LS643, LS644 and LS645 strains (Table S1). On the other hand, 1.5% (273) of all the probe-sets are uniquely present in LS643, LS644 and LS645 (Table S2) but absent in LS642. It is interesting to note that of all the unique probe-sets present in LS642 (Table S1) about 8% uniqueness is derived from phage sequences compared to about 1% phage sequences attributed to the uniqueness of LS643, LS644 and LS645 (Table S2). Such difference may indicate different ancestry of these two groups of IVb-v1 strains.

**Figure 4:**
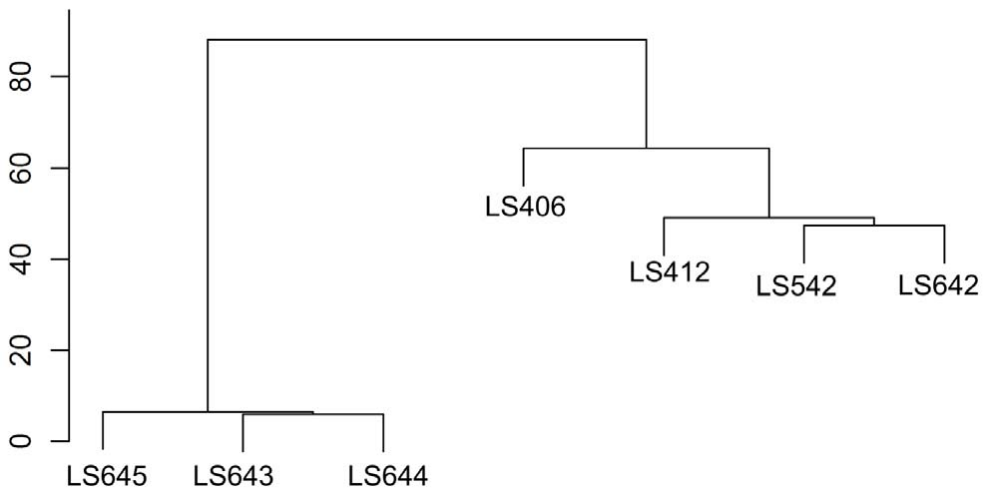
Hierarchical clustering based on the Robust Multi Array (RMA) analysis of the *L. monocytogenes* strains serotypes 4b (LS406, LS412) and IVb-v1 (LS542, LS642, LS643, LS644, LS645).

The microarray data was also used to investigate the genetic variability of the serotypes 4b and IVb-v1 strains using a Robust Multi Array (RMA) algorithm to assess the individual probe-set intensity without utilizing the mismatched-probe intensity information [33], [35], [36]. The hierarchical clustering (Fig. 4) based on the summarized probe-set intensity among the serotypes 4b and IVb-v1 strains were consistent with the MAS5.0 analysis for genetic contents (Fig. 3). The RMA analysis (Fig. 4) divides the seven strains into two groups. LS643, LS644 and LS645 formed a close cluster while the other four strains (LS406, LS412, LS542 and LS642) formed a separate group where the IVb-v1 strains (LS542 and LS642) and LS412 were much closer to each other than LS406. In term of pathophysiology it is interesting to note that the strains that were associated with invasive listeriosis (LS412, and LS642) were clustered closely while LS406, associated with a gastroenteritis outbreak [48], was branched away from this cluster.

**Figure 5:**
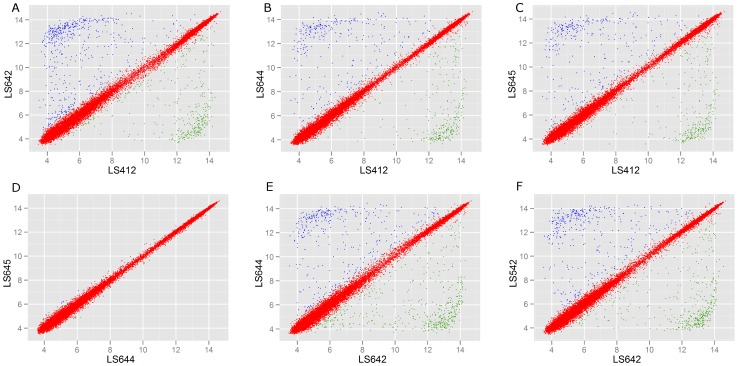
Scatter plots of the summarized Robust Multi-Array Averaging (RMA) intensities. A–C, between serotypes 4b (LS412) and IVb-v1 (LS642, A; LS644, B; LS645, C). D–E, between the same serotype, IVb-v1, isolated from Australia; from the different states (LS644 and LS645, D); from the same state (LS642 and LS644, E). F, between the same serotype, IVb-v1, isolated from the different countries (LS642; Australia and LS542; USA).

The variation of probe-set intensity (higher and lower than two-fold) between strains is a measure of pan-genomic variability that can be visually assessed by scatter plot. The plot also allows us to quantify the extent of variability and identify the genes that are different between the strains. In order to further assess the genomic differences between 4b and IVb-v1 strains and among IVb-v1 strains, we analyzed the scatter plots of various groups of strains (Fig. 5). Comparison of a serotype 4b strain, LS412, with the IVb-v1 strains (LS642, LS644 and LS645) revealed (Fig. 5-A, B, and C) that the numbers of probe-sets different between the paired strains out of a total of 18630 probe-sets analyzed are 846, 572 and 587 for LS642, LS644 and LS645, respectively. Similar analysis between LS412 and LS644 (Fig. 5B, n = 572) and between LS412 and LS645 (Fig. 5C, n = 587) revealed that 95–97% of probe-sets are identical, suggesting the close relationship between the two serotype IVb-v1 strains. In addition, the identical genetic make-up between LS644 and LS645 is also confirmed when a similar analysis was done between these two strains (Fig. 5D). The comparisons between LS642 and LS644 (Fig. 5E) and between LS642 and LS542 (Fig. 5F) revealed that there are 842 and 787 probe-sets different, respectively. These analyses clearly show the extent of genetic variability among the IVb-v1 strains. These results, however, are slightly different from the PFGE based clustering, possibly due to the differences in the amount of information between two methods. In addition, partial analysis of these Australian IVb-v1 strains based on the whole genome sequences [49] also revealed that LS542 and LS642 are more diverse from LS643, LS644 and 645 (data not shown) in agreement with other subtyping methods.

**Figure 6:**
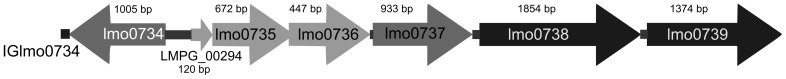
The organization of the genomic region containing the *lmo*0737 to *lmo0739* cassette that is present only in the *L. monocytogenes* serotypes IVb-v1 and 1/2a strains, confirmed by microarray analysis.

Our in-depth analysis of the extent of genetic variability of the IVb-v1 strains agrees with the previous findings that, although these strains share a common serotype IVb-v1, due to the presence of a serotype 1/2a specific locus *lmo*0737, the extent of genetic difference can be substantial. In order to investigate the extent of gene sharing between the serotype 1/2a and IVb-v1 strains, we explored the possibility of the existence of any other genomic footprints shared only by these IVb-v1 and serotype 1/2a strains. The comparison of the genetic contents of 84 strains in our microarray database clearly indicated that the serotype IVb-v1 strains are more closely related to the serotype 4b than to 1/2a. Serotypes IVb-v1 and 1/2a strains shared a total of ten unique probe-sets, representing two intergenic region and eight genes (Table 4). Multiple probe-sets analysis using *Listeria* GeneChip confirms that *lmo*0734– *lmo*0739 (Fig. 6) are present only in the *L. monocytogenes* serotypes IVb-v1 and 1/2a strains. Recently, Lee et al (2012) [22] reported the presence of the same gene cassette (*lmo*0734-*lmo*0739) in 23 serotype IVb-v1 strains. The total length of this region is approximately 6.3 kb. Two of these genes code for enzymes in pentose-phosphate pathway, one gene codes for an enzyme in the glycolysis/gluconeogenesis while *lmo*0734 codes for a *lacI* type transcription regulator and *lmo*0738 codes for a component of phosphotransferase system. At this point, we do not know the biological significance of these genes or how these genes were acquired by a set of 4b strains. A similar conclusion was also reached by Lee et al (2012) [22] from their study with 23 IVb-v1 strains collected in the US. Mutants with an in-frame deletion of this gene cassette in EGDe did not show any growth defect *in vitro* or *in vivo* [50] although these genes were up-regulated in the intestine of the infected mice and *lmo*0737 was down regulated in blood indicating the involvement during infection process [51]. Further experiments with isogenic constructs of IVb-v1 strains would be needed to understand the biological role of this cassette.

**Table 4:** Unique probe-sets in 1/2a and IVb-v1 *L. monocytogenes* strains.

		Serotype			
Probe-ID	Annotation (100% homology)	1/2a(n = 34)	1/2b(n = 20)	4b(n = 25)	IVb-v1(n = 5)
IGlmo0734_at	Intergenic region	+	−	−	+
IGlmo0735_x_at	Intergenic region	+	−	−	+
AARI_0596_s_at	LacI family-transcriptional regulator (lmo0734)	+	−	−	+
AARM_0745_S_at	LacI family-transcriptional regulator(lmo0734)	+	−	−	+
AARY_1549_s_at	6-phospho-beta-glucosidase (lmo0739)	+	−	−	+
lmo0735_s_at	Ribulose-5-Phosphate 3-Epimerase	+	−	−	+
lmo0736_at	Ribose 5-phosphate isomerase	+	−	−	+
lmo0737_s_at	Hypothetical protein	+	−	−	+
lmo0738_s_at	Phosphotransferase system (PTS) beta-glucoside-specificenzyme IIABC component	+	−	−	+
LMPG_00294_s_at	39 amino acid hypothetical protein (IGlmo0735)	+	−	−	+

Origin of this highly conserved region (LMOf2365_0734– LMOf2365_0739 cassette in IVb-v1 strains remains highly speculative at this point. Absence of this gene cassette in serotype 1/2b and majority of the serotype 4b strains and presence of this cassette in serotype 1/2a strains suggest horizontal gene transfer from serotype 1/2a to certain groups of serotype 4b strains [21], [22] although we could not locate any signs of phage genome or any transposon-like sequences flanking this region. It has been indicated that serotype 1/2a strains are more promiscuous in terms acquiring phage genomes [52]. It is conceivable that these 4b variant strains (IVb-v1) have some unique traits that make them more disposed to accepting genes from other organisms. Further experiments are however needed to prove or disprove this hypothesis. In addition, the analysis of this conserved region based on the whole genome sequence of these IVb-v1 strains [49] revealed that there are a few conserved SNPs between the 1/2a and IVb-v1 strains(data not shown) These nucleotide changes mostly resulted in silent mutations. However, there is a SNP in lmo0737, a hypothetical regulatory protein, resulting in a proline substituted serine in all four Australian strains. This change may be significant as the protein secondary structure could be altered leading to a different function of the protein.

One of the distinguishing features of the genomic contents of various epidemic clones of *L. monocytogenes* is the presence/absence of a restriction-modification (RM) cassette [53]. This cassette is characterized by the presence of a gene coding for Sau3A restriction enzyme (LMOf2365_0325), a DNA binding site (LMOf2365_0326) and a DNA methylase gene (LMOf2365_0327). Presence of these genes have been shown to be responsible for the resistance of the genomic DNA to Sau3A digestion while same genomic DNA remains sensitive to Mbo1 cleavage as cytosine methylation does not affect this enzyme. This RM gene cassette is present in all the ECI strains but absent in other 4b strains. Using the resistance/sensitivity to Sau3A and Mbo1 phenotype, Lee et al. [38] have shown that the collection of their IVb-v1 strains could be classified into two groups of which clonal groups 1 and 2 were sensitive to both Sau3A and Mbo1 while the third group was sensitive to Sau3A but resistant to Mbo1 indicating adenine rather than cytosine methylation of GATC sites in this group. As RM systems form the hallmark of bacterial genomic evolution, we decided to study the Sau3A and Mbo1 digestion pattern of the five IVb-v1 strains and also analyzed the presence/absence of the genes in the RM cassette region by microarray hybridization. Our results (Table 5) show that LS643, LS644 and LS645 genomic DNA were resistant to Sau3A digestion but sensitive to Mbo1 digestion indicating cytosine methylation at the GATC sites of these strains. This is also supported by the positive hybridization signals from the probe-sets representing LMOf2365_0325 (Sau3A enzyme), LMOf2365_0326 (DNA binding protein) and LMOf2365_0327 (DNA methylase) and also two downstream genes LMOf2365_0328 and LMOf2365_0329. The other two IVb-v1 strains, LS542 and LS642 were sensitive to both Sau3A and Mbo1 and were lacking genes from LMOf2365_0325 to LMOf2365_0328. The representative strains from 4b, 1/2a and 1/2b serotypes showed Mbo1 susceptibility while Sau3A results and presence/absence of the genes in the RM cassette were mixed (data not shown). The results further substantiated other genomic comparison data that LS643, LS644 and LS645 belonged to a genomic group while LS542 and LS642 belonged to separate groups. The results also showed that LS643, LS644 and LS645 represent a clonal group distinctly different from the three clonal groups of IVb-v1 strains described previously [22].

**Table 5:** Susceptibility to Mbo1 and Sau3A digestion and distribution of the genes in the restriction-modification cassette in selected *L. monocytogenes* strains.

						Probe ID			
Strain	Serotype	Mbo	Sau3A1	LMOf2365	LMOf2365	LMOf2365	LMOf2365	LMOf2365	LMOf2365
				0325_at	0326_at	0327_at	0328_s_at	IG0329_at	0329_s_at
LS412	4b	+	−	P	P	P	P	P	P
LS429	4b	+	+	A	A	A	A	A	P
LS406	4b	+	+	A	A	A	A	A	P
LS542	IVb-v1	+	+	A	A	A	A	A	P
LS642	IVb-v1	+	+	A	A	A	A	A	P
LS643	IVb-v1	+	−	P	P	P	P	P	P
LS644	IVb-v1	+	−	P	P	P	P	P	P
LS645	IVb-v1	+	−	P	P	P	P	P	P
LS146	1/2b	+	−	P	P	P	P	P	P
LS484	1/2b	+	+	A	A	A	A	A	A
LS686	1/2b	+	+	A	A	A	A	A	P
LS787	1/2a	+	−	A	A	A	P	P	P
LS120	1/2a	+	+	A	A	A	A	A	A

+: cut; − uncut; P: presence and A: absence of hybridization signals with the gene specific probe.

Dispersion of unique genotypes of *L. monocytogenes* throughout the world has been illustrated by the occurrence of multiple epidemic clones (EC) and multiple sequence types (ST) [54], [55]. The multilocus sequence typing (MLST) or simply sequence typing (ST) based subtyping has resulted in the formulation of clonal complex (CC), which unlike the ECs, does not require any epidemic outbreak association and thus represent a more overarching way to investigate the clonality of this organism. In several instances, ECs and CCs overlap with each other while in other cases they are distinct genogroups. For example all the known ECIs, ECIVs and ECIIs appear to fall under CC1, CC2, and CC6 respectively [55]. Using the draft whole genome sequences of the five IVb-v1 strains [49], we found that LS643, LS644 and LS645belong to ST240 while LS542 and LS642 belong to ST554 and ST572, respectively. The assignment of the same ST for LS643, LS644 and LS645 bolsters our previous assertions (Table 2, 3 and Figure 3) that these strains are very closely related to each other. A query of the MLST database at the Institute Pasteur (http://www.pasteur.fr/recherché/genopole/PF8/mlst/Lmono.html) revealed that the ST240 and ST693 are different by just one allele (*dapE*) and therefore by definition [56], these strains could be part of a clonal complex. Similarly, LS642 with ST572 match all but *dat* gene sequence with ST373 and thus could be part of a clonal group. It is interesting to note that LS643, LS644 and LS645 share ST240 with another IV-v1 strain isolated in 1959 in Switzerland from a human bacterimic patient [21].

In summary, our analyses clearly show that the IVb-v1 strains are genetically distinct from 4b strains and also among each other. These differences go beyond the presence/absence of a 6.3 kb DNA cassette as shown by microarray scatter plot analysis (Fig. 5) and by comparing the trees created among these strains with and without the 6.3 kb DNA specific probe-sets (data not shown). Three of the four human *L. monocytogenes* IVb-v1 strains from Australia shared a very extensive genetic homology indicating that these strains could be part of an outbreak cluster. These three strains also formed a different clonal group not reported previously [22]. The IVb-v1 pattern among geographically, temporally and genetically unrelated strains indicates that such variability can originate independently and the events are not of recent origin. The acquisition of approximately 6.3kb DNA from 1/2a serotype indicates that some 4b strains probably are more prone to genetic exchanges, a crucial requirement for emergence of newer traits.

## Supporting Information

Table S1 Probe-sets uniquely present in LS642 and absent in LS643, LS644 and LS645.
(DOCX)

Table S2 Probe-sets uniquely present in LS643, LS644 and LS645 but absent in LS642.
(DOCX)
