# Supplementary material for: Genomic Characterization of Novel Listeria monocytogenes Serotype 4b Variant Strains
Source: PLoS One. 2014 Feb 19;9(2):e89024. doi: 10.1371/journal.pone.0089024 (PMC3929640; doi:10.1371/journal.pone.0089024)
Supplement: Table S1 — Probe-sets uniquely present in LS642 and absent in LS643, LS644 and LS645. (DOCX) [file pone.0089024.s001.docx]

**Table S1: Probe-sets uniquely present in LS642 and absent in all LS643, LS644 and LS645**

| **Probe ID** | **Annotation** |
| --- | --- |
| AARI_0014_at | NK |
| AARI_0059_s_at | NK |
| AARI_0075_s_at | NK |
| AARI_0153_x_at | NK |
| AARI_0241_s_at | NK |
| AARI_0329_x_at | NK |
| AARI_0634_at | 100% similar to lmo1389 |
| AARK_0198_s_at | 99% similar to LMOf2365_0095 |
| AARK_0302_x_at | NK |
| AARK_1335_at | 99% similar to LMOf2365_2040 |
| AARK_1658_s_at | 100% similar to LMOf2365_1621 |
| AARK_1750_s_at | NK |
| AARL_0164_at | NK |
| AARL_0363_s_at | NK |
| AARL_0461_s_at | NK |
| AARL_0848_at | NK |
| AARL_0854_s_at | 98% similar to LMHCC_1920 |
| AARM_0084_s_at | 98% similar to LMHCC_2618 |
| AARM_0374_x_at | NK |
| AARM_0510_s_at | 100% similar to lmo1827 |
| AARM_1320_x_at | NK |
| AARM_1690_at | 98% similar to LMHCC_2257 |
| AARM_1763_s_at | 99% similar to lmo0509 |
| AARM_1775_at | NK |
| AARO_0115_x_at | NK |
| AARO_1260_at | NK |
| AARO_1260_x_at | NK |
| AARO_1401_x_at | 98% similar to LMOf2365_0507 |
| AARO_1755_s_at | NK |
| AARY_0634_at | 100% similar to lmo0776 |
| AARY_0964_s_at | 100% similar to lmo2591 |
| AARY_1347_x_at | 100% similar to lmo2751 |
| AARY_1531_s_at | 99% similar to lmo0007 |
| IGLm4b_00330_x_at | intergenic region |
| IGLm4b_00400_x_at | intergenic region |
| IGLm4b_01145_x_at | intergenic region |
| IGLm4b_01910_x_at | intergenic region |
| IGLm4b_02039_x_at | intergenic region |
| IGLMHCC_0028_at | intergenic region |
| IGLMHCC_0028_x_at | intergenic region |
| IGLMHCC_0060_at | intergenic region |
| IGLMHCC_0060_x_at | intergenic region |
| IGLMHCC_0253_x_at | intergenic region |
| IGLMHCC_0339_at | intergenic region |
| IGLMHCC_0401_at | intergenic region |
| IGLMHCC_0840_at | intergenic region |
| IGLMHCC_0840_x_at | intergenic region |
| IGLMHCC_0977_at | intergenic region |
| IGLMHCC_1400_s_at | intergenic region |
| IGLMHCC_1402_s_at | intergenic region |
| IGLMHCC_1403_s_at | intergenic region |
| IGLMHCC_1579_at | intergenic region |
| IGLMHCC_1818_at | intergenic region |
| IGLMHCC_2003_x_at | intergenic region |
| IGLMHCC_2079_at | intergenic region |
| IGLMHCC_2182_at | intergenic region |
| IGLMHCC_2255_at | intergenic region |
| IGLMHCC_2259_at | intergenic region |
| IGLMHCC_2259_x_at | intergenic region |
| IGLMHCC_2304_at | intergenic region |
| IGLMHCC_2633_at | intergenic region |
| IGLMHCC_2645_at | intergenic region |
| IGLMHCC_2694_x_at | intergenic region |
| IGLMHCC_2713_x_at | intergenic region |
| IGLMHCC_2864_at | intergenic region |
| IGLMHCC_2899_at | intergenic region |
| IGLMHCC_2940_s_at | intergenic region |
| IGLMHCC_2942_s_at | intergenic region |
| IGLMHCC_2950_s_at | intergenic region |
| IGLMHCC_2972_x_at | intergenic region |
| IGLMHCC_2973_x_at | intergenic region |
| IGLMHCC_2983_s_at | intergenic region |
| IGLMHCC_3000_at | intergenic region |
| IGLMHCC_3009_at | intergenic region |
| IGlmo0382_s_at | intergenic region |
| IGlmo0487_x_at | intergenic region |
| IGlmo0772_at | intergenic region |
| IGlmo1133_x_at | intergenic region |
| IGlmo1263_x_at | intergenic region |
| IGlmo1452_at | intergenic region |
| IGlmo1461_x_at | intergenic region |
| IGlmo1614_x_at | intergenic region |
| IGlmo1686_x_at | intergenic region |
| IGlmo1997_x_at | intergenic region |
| IGlmo2173_x_at | intergenic region |
| IGlmo2324_at | intergenic region |
| IGlmo2324_x_at | intergenic region |
| IGlmo2328_x_at | intergenic region |
| IGlmo2330_at | intergenic region |
| IGlmo2330_x_at | intergenic region |
| IGlmo2595_at | intergenic region |
| IGlmo2596_at | intergenic region |
| IGlmo2596_x_at | intergenic region |
| IGLMOf2365_0163_at | intergenic region |
| IGLMOf2365_0163_s_at | intergenic region |
| IGLMOf2365_0163_x_at | intergenic region |
| IGLMOf2365_0164_x_at | intergenic region |
| IGLMOf2365_0165_at | intergenic region |
| IGLMOf2365_0167_at | intergenic region |
| IGLMOf2365_0348_s_at | intergenic region |
| IGLMOf2365_0350_s_at | intergenic region |
| IGLMOf2365_0356_s_at | intergenic region |
| IGLMOf2365_0357_s_at | intergenic region |
| IGLMOf2365_0358_s_at | intergenic region |
| IGLMOf2365_0359_s_at | intergenic region |
| IGLMOf2365_0361_s_at | intergenic region |
| IGLMOf2365_0499_x_at | intergenic region |
| IGLMOf2365_0507_at | intergenic region |
| IGLMOf2365_0507_x_at | intergenic region |
| IGLMOf2365_0508_s_at | intergenic region |
| IGLMOf2365_0550_x_at | intergenic region |
| IGLMOf2365_0687_at | intergenic region |
| IGLMOf2365_0688_at | intergenic region |
| IGLMOf2365_0771_s_at | intergenic region |
| IGLMOf2365_2056_s_at | intergenic region |
| IGLMOf2365_2057_x_at | intergenic region |
| IGLMOf2365_2237_at | intergenic region |
| Lm4b_00011_s_at | Putative mevalonate diphosphate decarboxylase/GI=225875112 |
| Lm4b_00387_x_at | Putative different proteins/GI=225875457 |
| Lm4b_00627_s_at | Putative cell surface protein/GI=225875692 |
| Lm4b_00828_s_at | Putative fructokinase/GI=225875892 |
| Lm4b_01134_at | Putative ABC transporter, ATP-binding protein/GI=225876193 |
| Lm4b_02566_s_at | Putative cobalt ABC transporter, permease protein/GI=225877607 |
| LMBG_00598_s_at | conserved hypothetical protein |
| LMBG_01625_s_at | conserved hypothetical protein |
| LMBG_01654_at | phage protein |
| LMBG_01654_x_at | phage protein |
| LMBG_01670_x_at | phage protein |
| LMBG_02287_s_at | conserved hypothetical protein/Pfam=PF06736.3 |
| LMBG_02493_at | conserved hypothetical protein |
| LMBG_02494_at | conserved hypothetical protein/Pfam=PF09346.2 |
| LMBG_02495_at | conserved hypothetical protein |
| LMBG_02496_s_at | conserved hypothetical protein |
| LMBG_02741_at | conserved hypothetical protein/Pfam=PF09479.2 |
| LMBG_02741_s_at | conserved hypothetical protein/Pfam=PF09479.2 |
| LMBG_02741_x_at | conserved hypothetical protein/Pfam=PF09479.2 |
| LMBG_02816_s_at | glycosyl transferase/Pfam=PF03636.7 |
| LMBG_02884_x_at | conserved hypothetical protein |
| LMBG_02989_s_at | predicted protein |
| LMFG_01139_at | conserved hypothetical protein/Pfam=PF04241.7 |
| LMFG_02381_s_at | FtsKSpoIIIE family protein/Pfam=PF01580.10 |
| LMFG_02382_at | conserved hypothetical protein |
| LMFG_02383_at | conserved hypothetical protein |
| LMFG_02384_s_at | conserved hypothetical protein |
| LMFG_02385_at | conserved hypothetical protein |
| LMFG_02386_at | conserved hypothetical protein |
| LMFG_02399_s_at | phage integrase/Pfam=PF00589.14 |
| LMFG_02401_s_at | conserved hypothetical protein/Pfam=PF01381.14 |
| LMFG_02402_at | predicted protein/Pfam=PF01381.14 |
| LMFG_02676_x_at | phage protein |
| LMFG_02983_s_at | phage protein |
| LMFG_02991_at | major capsid protein |
| LMFG_02992_at | phage protein |
| LMFG_02992_x_at | phage protein |
| LMFG_02993_x_at | phage protein |
| LMFG_03169_s_at | internalin A/Pfam=PF09479.2 |
| LMFG_03204_s_at | phage protein/Pfam=PF00376.15 |
| LMFG_03220_x_at | predicted protein |
| LMFG_03228_s_at | cell wall surface anchor family protein/Pfam=PF08191.3 |
| LMHCC_1005_at | mutM formamidopyrimidine-DNA glycosylase/GI=217333559 |
| LMHCC_1125_s_at | foldase protein PrsA/GI=217333679 |
| LMHCC_1356_s_at | conserved hypothetical protein/GI=217333907 |
| LMHCC_1398_at | hypothetical protein/GI=217333949 |
| LMHCC_1399_at | conserved hypothetical protein/GI=217333950 |
| LMHCC_1399_x_at | conserved hypothetical protein/GI=217333950 |
| LMHCC_1400_s_at | conserved hypothetical protein/GI=217333951 |
| LMHCC_1401_s_at | conserved hypothetical protein/GI=217333952 |
| LMHCC_1402_s_at | cytosine-specific methyltransferase/GI=217333953 |
| LMHCC_1444_s_at | cbiO cobalt import ATP-binding protein CbiO/GI=217333995 |
| LMHCC_1817_s_at | transcriptional regulator/GI=217334364 |
| LMHCC_2009_x_at | domain of unknown function, putative/GI=217334554 |
| LMHCC_2112_x_at | conserved hypothetical protein/GI=217334656 |
| LMHCC_2134_s_at | alcohol dehydrogenase, zinc-dependent/GI=217334678 |
| LMHCC_2255_s_at | conserved hypothetical protein/GI=217334799 |
| LMHCC_2256_at | conserved hypothetical protein/GI=217334800 |
| LMHCC_2282_s_at | transcriptional regulator, DeoR family/GI=217334826 |
| LMHCC_2303_s_at | IspD/GI=217334847 |
| LMHCC_2333_s_at | PRDPTS system IIA 2 domain regulatory protein/GI=217334876 |
| LMHCC_2726_s_at | pts system mannitol-specific eiicba component (eiicba-mtl) (eii-mtl)/GI=217335267 |
| LMHCC_2753_s_at | gshAB glutamate--cysteine ligasegamma-glutamylcysteine synthetase/GI=217335294 |
| LMHCC_2946_s_at | conserved hypothetical protein/GI=217335483 |
| LMHCC_2959_x_at | conserved hypothetical protein/GI=217335496 |
| LMHCC_2962_at | conserved hypothetical protein/GI=217335499 |
| LMHCC_2963_at | hypothetical protein/GI=217335500 |
| LMHCC_2963_x_at | hypothetical protein/GI=217335500 |
| LMHCC_2964_at | conserved hypothetical protein/GI=217335501 |
| LMHCC_2965_at | hypothetical protein/GI=217335502 |
| LMHCC_2965_s_at | hypothetical protein/GI=217335502 |
| LMHCC_2965_x_at | hypothetical protein/GI=217335502 |
| LMHCC_2966_at | conserved domain protein/GI=217335503 |
| LMHCC_2967_at | hypothetical protein/GI=217335504 |
| LMHCC_2968_at | putative gp69/GI=217335505 |
| LMHCC_2968_x_at | putative gp69/GI=217335505 |
| LMHCC_2969_s_at | gp59/GI=217335506 |
| LMHCC_2969_x_at | gp59/GI=217335506 |
| LMHCC_2972_x_at | conserved domain protein/GI=217335509 |
| LMHCC_2974_s_at | gp59/GI=217335511 |
| LMHCC_2974_x_at | gp59/GI=217335511 |
| LMHCC_2977_s_at | protein gp66/GI=217335514 |
| LMHCC_2983_at | putative scaffolding protein/GI=217335520 |
| LMHCC_2983_s_at | putative scaffolding protein/GI=217335520 |
| LMHCC_2983_x_at | putative scaffolding protein/GI=217335520 |
| LMHCC_2985_s_at | protein gp8/GI=217335522 |
| LMHCC_2995_at | putative gp18/GI=217335532 |
| LMHCC_3000_at | putative gp17-1 protein/GI=217335537 |
| LMHCC_3001_at | Gp18 protein/GI=217335538 |
| LMHCC_3005_s_at | lipoprotein, putative/GI=217335542 |
| LMHCC_3008_x_at | morphine 6-dehydrogenase (Naloxone reductase)/GI=217335545 |
| LMHG_00244_x_at | methylatedDNAproteincysteine methyltransferase/Pfam=PF01035.12 |
| LMHG_00285_at | transcriptional regulator/Pfam=PF01047.14 |
| LMHG_00422_s_at | ABC transporter/Pfam=PF00664.15 |
| LMHG_00428_x_at | multidrug efflux transporter transcriptional regulatory protein/Pfam=PF00376.15 |
| LMHG_00803_x_at | morphine 6dehydrogenase/Pfam=PF00248.13 |
| LMHG_02364_x_at | conserved hypothetical protein/Pfam=PF07885.8 |
| LMHG_02367_x_at | copper resistance domaincontaining protein/Pfam=PF05425.5 |
| LMHG_03171_x_at | cobalamin biosynthesis protein CbiG |
| LMHG_03198_s_at | conserved hypothetical protein |
| LMIG_02087_x_at | conserved hypothetical protein |
| LMIG_02452_x_at | conserved hypothetical protein/Pfam=PF03992.8 |
| LMIG_02904_s_at | parC/Pfam=PF00521.12 |
| LMJG_01498_at | predicted protein |
| LMJG_02938_s_at | phage protein |
| LMKG_02524_x_at | conserved hypothetical protein |
| LMKG_02849_at | predicted protein |
| LMKG_02849_s_at | predicted protein |
| LMKG_02849_x_at | predicted protein |
| LMKG_02912_at | predicted protein |
| LMKG_02912_x_at | predicted protein |
| LMKG_02914_at | predicted protein |
| LMKG_02915_at | conserved hypothetical protein |
| LMKG_02915_s_at | conserved hypothetical protein |
| LMLG_00210_x_at | conserved hypothetical protein |
| LMLG_00921_x_at | PTS system/Pfam=PF02255.8 |
| LMLG_00992_at | glutamate 5kinase/Pfam=PF00696.20 |
| LMLG_01405_x_at | PTS system/Pfam=PF02255.8 |
| LMLG_02127_at | conserved hypothetical protein/Pfam=PF03422.7 |
| LMMG_03068_x_at | conserved hypothetical protein |
| lmo0216_x_at | GI=16409581 |
| lmo0302_s_at | GI=16409667 |
| lmo0335_x_at | GI=16409713 |
| lmo1358_x_at | GI=16410774 |
| lmo1517_s_at | GI=16410946 |
| lmo2288_s_at | Protein gp15 [Bacteriophage A118]/GI=16411758 |
| lmo2290_s_at | Portein gp13 [Bacteriophage A118]/GI=16411760 |
| lmo2304_s_at | Bacteriophage A118 gp65 protein/GI=16411774 |
| lmo2317_s_at | GI=16411787 |
| lmo2319_s_at | GI=16411789 |
| lmo2320_at | GI=16411790 |
| lmo2320_s_at | GI=16411790 |
| lmo2324_s_at | GI=16411813 |
| lmo2325_at | GI=16411814 |
| lmo2326_s_at | GI=16411815 |
| lmo2330_s_at | GI=16411819 |
| lmo2331_s_at | GI=16411820 |
| lmo2406_s_at | GI=16411894 |
| lmo2569_s_at | GI=16412057 |
| lmo2594_s_at | GI=16412082 |
| LMOf2365_0088_s_at | hypothetical protein/GI=46879574 |
| LMOf2365_0155_s_at | oligopeptide ABC transporter, permease protein/GI=46879641 |
| LMOf2365_0163_s_at | hypothetical protein/GI=46879648 |
| LMOf2365_0164_s_at | hypothetical protein/GI=46879649 |
| LMOf2365_0165_s_at | hypothetical protein/GI=46879650 |
| LMOf2365_0166_s_at | hypothetical protein/GI=46879651 |
| LMOf2365_0348_s_at | hypothetical protein/GI=46879833 |
| LMOf2365_0348_x_at | hypothetical protein/GI=46879833 |
| LMOf2365_0354_s_at | conserved domain protein/GI=46879839 |
| LMOf2365_0355_at | conserved hypothetical protein/GI=46879840 |
| LMOf2365_0356_s_at | hypothetical protein/GI=46879841 |
| LMOf2365_0357_s_at | conserved hypothetical protein/GI=46879842 |
| LMOf2365_0358_s_at | hypothetical protein/GI=46879843 |
| LMOf2365_0359_s_at | hypothetical protein/GI=46879844 |
| LMOf2365_0360_s_at | hypothetical protein/GI=46879845 |
| LMOf2365_0551_s_at | phosphosugar-binding transcriptional regulator, RpiR family/GI=46880032 |
| LMOf2365_0566_s_at | putative N-carbamoyl-L-amino acid amidohydrolase/GI=46880048 |
| LMOf2365_0658_x_at | hydrolase, isochorismatase family/GI=46880140 |
| LMOf2365_0674_x_at | putative transaldolase/GI=46880155 |
| LMOf2365_0687_at | conserved hypothetical protein/GI=46880168 |
| LMOf2365_0704_s_at | oxidoreductase, short-chain dehydrogenasereductase family/GI=46880186 |
| LMOf2365_0729_s_at | flagellar motor switch domain protein/GI=46880210 |
| LMOf2365_0747_s_at | flgC flagellar basal-body rod protein FlgC/GI=46880228 |
| LMOf2365_0784_s_at | conserved hypothetical protein/GI=46880265 |
| LMOf2365_0828_x_at | HD domain protein/GI=46880308 |
| LMOf2365_0831_s_at | conserved hypothetical protein/GI=46880311 |
| LMOf2365_0832_s_at | conserved hypothetical protein/GI=46880312 |
| LMOf2365_0844_s_at | hydroxymethylglutaryl-CoA reductase, degradative/GI=46880324 |
| LMOf2365_1004_at | bsaA glutathione peroxidase/GI=46880483 |
| LMOf2365_1452_s_at | pyridine nucleotide-disulfide oxidoreductase family protein/GI=46880929 |
| LMOf2365_1776_s_at | RNA methyltransferase, TrmA family/GI=46881251 |
| LMOf2365_1880_s_at | heavy metal-binding protein/GI=46881354 |
| LMOf2365_1898_s_at | conserved hypothetical protein/GI=46881371 |
| LMOf2365_2172_s_at | putative transporter/GI=46881644 |
| LMOf2365_2690_s_at | conserved hypothetical protein/GI=46882162 |
| LMOf6854_2351_at | protein gp14/GI=47015588 |
| LMOf6854_2351_s_at | protein gp14/GI=47015588 |
| LMOf6854_2351_x_at | protein gp14/GI=47015588 |
| LMOf6854_2352_s_at | major tail protein, putative/GI=47015589 |
| LMOf6854_2355_at | protein gp10/GI=47015592 |
| LMOf6854_2356_at | gp9/GI=47015593 |
| LMOf6854_2361_at | gp4/GI=47015598 |
| LMOf6854_2361_x_at | gp4/GI=47015598 |
| LMOf6854_2362_s_at | phage portal protein, putative, A118 family/GI=47015599 |
| LMOf6854_2363_at | phage terminase, large subunit, PBSX family/GI=47015600 |
| LMOf6854_2370_s_at | conserved hypothetical protein/GI=47015607 |
| LMOG_00600_s_at | predicted protein |
| LMOG_01467_s_at | predicted protein |
| LMOG_02726_at | predicted protein |
| LMOG_03105_s_at | Gp32 protein |
| LMOG_03122_s_at | conserved hypothetical protein/Pfam=PF05126.4 |
| LMOG_03149_at | predicted protein |
| LMOh7858_0084_at | hypothetical protein/GI=47019399 |
| LMOh7858_0085_s_at | hypothetical protein/GI=47019400 |
| LMOh7858_0086_at | synthetic lethal KAR3-like protein/GI=47019401 |
| LMOh7858_0087_at | conserved hypothetical protein/GI=47019402 |
| LMOh7858_0088_at | conserved hypothetical protein/GI=47019403 |
| LMOh7858_0088_s_at | conserved hypothetical protein/GI=47019403 |
| LMOh7858_0089_s_at | conserved hypothetical protein/GI=47019404 |
| LMOh7858_0090_at | hypothetical protein/GI=47019405 |
| LMOh7858_0090_s_at | hypothetical protein/GI=47019405 |
| LMOh7858_0090_x_at | hypothetical protein/GI=47019405 |
| LMOh7858_0091_at | conserved hypothetical protein/GI=47019406 |
| LMOh7858_0092_s_at | conserved hypothetical protein/GI=47019407 |
| LMOh7858_0093_at | conserved hypothetical protein/GI=47019408 |
| LMOh7858_0097_at | hypothetical protein/GI=47019412 |
| LMOh7858_0097_x_at | hypothetical protein/GI=47019412 |
| LMOh7858_0165_x_at | conserved hypothetical protein/GI=47017599 |
| LMOh7858_0166_x_at | conserved hypothetical protein/GI=47017600 |
| LMOh7858_0368_at | hypothetical protein/GI=47020242 |
| LMOh7858_0368_x_at | hypothetical protein/GI=47020242 |
| LMOh7858_0500_at | conserved hypothetical protein/GI=47019144 |
| LMOh7858_0512_at | conserved hypothetical protein/GI=47019108 |
| LMOh7858_0512_x_at | conserved hypothetical protein/GI=47019108 |
| LMOh7858_0532_at | HD domain protein/GI=47019128 |
| LMOh7858_0532_x_at | HD domain protein/GI=47019128 |
| LMOh7858_0662_at | conserved hypothetical protein/GI=47017234 |
| LMOh7858_0719_at | transcriptional activator, putative/GI=47019937 |
| LMOh7858_0862_x_at | spermidineputrescine ABC transporter, spermidineputrescine-binding protein/GI=47017691 |
| LMOh7858_0863_at | oxidoreductase, short chain dehydrogenasereductase family/GI=47017692 |
| LMOh7858_0864_at | merR transcriptional regulator, MerR family/GI=47017693 |
| LMOh7858_0864_x_at | merR transcriptional regulator, MerR family/GI=47017693 |
| LMOh7858_0865_at | conserved hypothetical protein/GI=47017694 |
| LMOh7858_0866_at | transcription regulator/GI=47017695 |
| LMOh7858_0867_at | RNA-directed DNA polymerase from retron ec67/GI=47017696 |
| LMOh7858_0867_s_at | RNA-directed DNA polymerase from retron ec67/GI=47017696 |
| LMOh7858_0870_s_at | HD domain protein/GI=47017728 |
| LMOh7858_1161_at | Helix-turn-helix domain protein/GI=47019609 |
| LMOh7858_1162_at | transcriptional regulator, putative/GI=47019610 |
| LMOh7858_1163_at | site-specific recombinase, phage integrase family, putative/GI=47019611 |
| LMOh7858_1164_at | nikA protein, putative/GI=47019612 |
| LMOh7858_1165_at | hypothetical protein/GI=47019744 |
| LMOh7858_1166_at | conserved hypothetical protein/GI=47019613 |
| LMOh7858_1167_at | Rlx-like protein/GI=47019745 |
| LMOh7858_1168_at | AAA superfamily ATPase/GI=47019614 |
| LMOh7858_1169_at | hypothetical protein/GI=47019615 |
| LMOh7858_1170_at | putative immunity repressor protein/GI=47019616 |
| LMOh7858_1171_at | hypothetical protein/GI=47019617 |
| LMOh7858_1172_at | hypothetical protein/GI=47019750 |
| LMOh7858_1173_at | hypothetical protein/GI=47019618 |
| LMOh7858_1174_at | conserved hypothetical protein/GI=47019619 |
| LMOh7858_1200_at | conserved hypothetical protein/GI=47019644 |
| LMOh7858_1203_x_at | conserved hypothetical protein/GI=47019647 |
| LMOh7858_1495_s_at | conserved hypothetical protein/GI=47018236 |
| LMOh7858_2753_at | hypothetical protein/GI=47018085 |
| LMOh7858_2754_at | ulcer associated adenine specific DNA methyltransferase, putative/GI=47018086 |
| LMOh7858_2755_at | conserved hypothetical protein/GI=47018105 |
| LMOh7858_2756_at | DNA repair protein RadC/GI=47018087 |
| LMOh7858_2757_at | hypothetical protein/GI=47018088 |
| LMOh7858_2758_at | hypothetical protein/GI=47018089 |
| LMOh7858_2759_at | hypothetical protein/GI=47018090 |
| LMOh7858_2760_at | hypothetical protein/GI=47018091 |
| LMOh7858_2761_at | hypothetical protein/GI=47018092 |
| LMOh7858_2762_at | hypothetical protein/GI=47018093 |
| LMOh7858_2763_at | hypothetical protein/GI=47018094 |
| LMOh7858_2764_at | site-specific recombinase, phage integrase family, putative/GI=47018095 |
| LMOh7858_2958_at | dihydroxyacetone kinase family protein/GI=47019295 |
| LMOh7858_3069_s_at | conserved hypothetical protein/GI=47018361 |
| LMOh7858_3070_s_at | conserved hypothetical protein/GI=47018340 |
| LMOh7858_3083_s_at | peptidase, M20M25M40 family/GI=47018352 |
| LMRG_00650_x_at | cbiM/Pfam=PF01891.8 |
| LMRG_01514_at | phage protein/Pfam=PF01381.14 |
| LMRG_01538_at | phage protein |
| LMRG_01538_s_at | phage protein |
| LMRG_01538_x_at | phage protein |
| LMRG_01541_at | major tail shaft protein |
| LMRG_01541_x_at | major tail shaft protein |
| LMRG_01773_s_at | phosphoglucomutasephosphomannomutase family protein/Pfam=PF02880.8 |
| LMRG_02394_x_at | predicted protein |
| LMRG_02918_x_at | predicted protein |
| LMSG_00004_at | phage protein/Pfam=PF04404.4 |
| LMSG_00005_at | phage protein/Pfam=PF05565.3 |
| LMSG_00005_x_at | phage protein/Pfam=PF05565.3 |
| LMSG_01253_x_at | conserved hypothetical protein/Pfam=PF00376.15 |
| LMSG_01402_at | nitroreductase/Pfam=PF00881.16 |
| LMSG_01821_s_at | conserved hypothetical protein/Pfam=PF05043.5 |
| LMSG_02556_x_at | conserved hypothetical protein |
| LMSG_02949_at | phage integrase/Pfam=PF00589.14 |
| LMSG_02950_x_at | TerS/Pfam=PF03592.8 |
| LMSG_02954_at | phage protein/Pfam=PF06356.3 |
| LMSG_02968_s_at | phage protein |
| LMSG_03005_at | major tail shaft protein |
| LMSG_03005_x_at | major tail shaft protein |
| LMSG_03010_at | conserved hypothetical protein |
| LMSG_03010_x_at | conserved hypothetical protein |
| LMSG_03012_at | conserved hypothetical protein |
| LMSG_03013_at | conserved hypothetical protein |
| LMSG_03013_x_at | conserved hypothetical protein |
| LMSG_03142_x_at | phage protein |
| LMSG_03145_at | predicted protein |
| LMSG_03146_x_at | predicted protein |
| LMSG_03157_at | predicted protein |
| LMSG_03157_x_at | predicted protein |
| LMSG_03164_x_at | conserved hypothetical protein |
| LMSG_03170_at | phage protein |
| LMSG_03171_s_at | phage protein |
| LMSG_03174_s_at | phage protein/Pfam=PF03374.6 |
| LMSG_03174_x_at | phage protein/Pfam=PF03374.6 |

NK: Not known
