# Supplementary material for: Genomic Characterization of Novel Listeria monocytogenes Serotype 4b Variant Strains
Source: PLoS One. 2014 Feb 19;9(2):e89024. doi: 10.1371/journal.pone.0089024 (PMC3929640; doi:10.1371/journal.pone.0089024)
Supplement: Table S2 — Probe-sets uniquely present in LS643, LS644 and LS645 but absent in LS642. (DOCX) [file pone.0089024.s002.docx]

**Table S2: Probe-sets uniquely present in all LS643, LS644 and LS645 but absent in LS642**

| **Probe ID** | **Annotation** |
| --- | --- |
| AARI_0390_at | 100% similar to lmo0762 |
| AARI_0569_at | 99% similar to lmo0333 |
| AARI_0697_s_at | 100% similar to lmo0707 |
| AARK_0852_x_at | NK |
| AARK_0871_s_at | NK |
| AARK_1188_at | 99% similar to LMOf2365_0508 |
| AARL_0232_s_at | NK |
| AARL_0233_s_at | 98% similar to LMOf2365_0325 |
| AARL_0357_s_at | NK |
| AARL_0509_s_at | NK |
| AARL_0557_x_at | NK |
| AARL_0581_at | 99% similar to LMHCC_2781 |
| AARM_0199_at | 100% similar to lmo0333 |
| AARM_0327_x_at | NK |
| AARM_0341_x_at | NK |
| AARM_1611_s_at | 99% similar to lmo0723 |
| AARM_1674_s_at | 99% similar to lmo1096 |
| AARM_1698_x_at | 99% similar to lmo1867 |
| AARM_1710_x_at | 100% similar to lmo1012 |
| AARO_1258_s_at | NK |
| AARO_1259_s_at | NK |
| AARO_1920_x_at | 98% similar to lmo0463 |
| AARO_1924_s_at | 100% similar to LMOf2365_1684 |
| AARY_0504_at | NK |
| AARY_0650_s_at | 100% similar to lmo0601 |
| AARY_0939_at | NK |
| AARY_0939_x_at | NK |
| AARY_1250_at | 100% similar to lmo0812 |
| AARY_1609_s_at | 100% similar to lmo1729 |
| IGLm4b_00079_at | intergenic region |
| IGLm4b_00080a_at | intergenic region |
| IGLm4b_00081_x_at | intergenic region |
| IGLm4b_00150_at | intergenic region |
| IGLm4b_00150_x_at | intergenic region |
| IGLm4b_00152_at | intergenic region |
| IGLm4b_00496_at | intergenic region |
| IGLm4b_00496_x_at | intergenic region |
| IGLm4b_00497_at | intergenic region |
| IGLm4b_00497_x_at | intergenic region |
| IGLm4b_00499_s_at | intergenic region |
| IGLm4b_00499_x_at | intergenic region |
| IGLm4b_00543_at | intergenic region |
| IGLm4b_00547_at | intergenic region |
| IGLm4b_00548_at | intergenic region |
| IGLm4b_00677_at | intergenic region |
| IGLm4b_00677_x_at | intergenic region |
| IGLm4b_01609_s_at | intergenic region |
| IGLm4b_02038_at | intergenic region |
| IGLm4b_02138_x_at | intergenic region |
| IGLm4b_02231_at | intergenic region |
| IGLm4b_02231_x_at | intergenic region |
| IGLm4b_02562_s_at | intergenic region |
| IGLm4b_02562_x_at | intergenic region |
| IGLMHCC_0157_x_at | intergenic region |
| IGLMHCC_0176_x_at | intergenic region |
| IGLMHCC_0502_x_at | intergenic region |
| IGLMHCC_0889_x_at | intergenic region |
| IGLMHCC_0953_x_at | intergenic region |
| IGLMHCC_1085_x_at | intergenic region |
| IGLMHCC_1178_x_at | intergenic region |
| IGLMHCC_1323_at | intergenic region |
| IGLMHCC_1542_x_at | intergenic region |
| IGLMHCC_1608_at | intergenic region |
| IGLMHCC_1883_at | intergenic region |
| IGLMHCC_1978_at | intergenic region |
| IGLMHCC_2165_x_at | intergenic region |
| IGLMHCC_2265_s_at | intergenic region |
| IGLMHCC_2355_at | intergenic region |
| IGLMHCC_2490_s_at | intergenic region |
| IGLMHCC_2719_x_at | intergenic region |
| IGlmo0149_s_at | intergenic region |
| IGlmo0149_x_at | intergenic region |
| IGlmo0464_x_at | intergenic region |
| IGlmo0570_x_at | intergenic region |
| IGlmo0746_s_at | intergenic region |
| IGlmo0746_x_at | intergenic region |
| IGlmo0753_x_at | intergenic region |
| IGlmo1597_at | intergenic region |
| IGlmo1598_x_at | intergenic region |
| IGlmo1722_x_at | intergenic region |
| IGlmo2206_x_at | intergenic region |
| IGlmo2806_x_at | intergenic region |
| IGLMOf2365_0092_x_at | intergenic region |
| IGLMOf2365_0325_at | intergenic region |
| IGLMOf2365_0326_at | intergenic region |
| IGLMOf2365_0328_at | intergenic region |
| IGLMOf2365_0329_at | intergenic region |
| IGLMOf2365_0382_s_at | intergenic region |
| IGLMOf2365_0394_x_at | intergenic region |
| IGLMOf2365_0625_at | intergenic region |
| IGLMOf2365_0776_at | intergenic region |
| IGLMOf2365_0776_x_at | intergenic region |
| IGLMOf2365_0777_s_at | intergenic region |
| IGLMOf2365_0790_at | intergenic region |
| IGLMOf2365_0795_at | intergenic region |
| IGLMOf2365_1139_at | intergenic region |
| IGLMOf2365_1471_at | intergenic region |
| IGLMOf2365_1702_x_at | intergenic region |
| IGLMOf2365_1750_at | intergenic region |
| IGLMOf2365_1750_x_at | intergenic region |
| IGLMOf2365_2052_x_at | intergenic region |
| IGLMOf2365_2511_at | intergenic region |
| IGLMOf2365_2511_x_at | intergenic region |
| IGLMOf2365_2542_at | intergenic region |
| IGLMOf2365_2542_x_at | intergenic region |
| IGLMOf2365_2565_s_at | intergenic region |
| IGLMOf2365_2568_x_at | intergenic region |
| IGLMOf2365_2631_at | intergenic region |
| IGLMOf2365_2632_s_at | intergenic region |
| IGLMOf2365_2750_at | intergenic region |
| IGLMOf2365_2750_x_at | intergenic region |
| IGLMOf2365_2796_x_at | intergenic region |
| IGLMOf2365_2872_at | intergenic region |
| Lm4b_00070_x_at | Putative DNA segregation ATPase FtsKSpoIIIE/GI=225875168 |
| Lm4b_00071_s_at | Putative hypothetical protein/GI=225875169 |
| Lm4b_00072_s_at | Hypothetical protein of unknown function/GI=225875170 |
| Lm4b_00073_s_at | Hypothetical protein of unknown function/GI=225875171 |
| Lm4b_00080a_s_at | Hypothetical protein of unknown function |
| Lm4b_00080b_s_at | Hypothetical protein of unknown function |
| Lm4b_00149_at | Hypothetical protein of unknown function/GI=225875246 |
| Lm4b_00149_x_at | Hypothetical protein of unknown function/GI=225875246 |
| Lm4b_00150_s_at | Hypothetical protein of unknown function |
| Lm4b_00370_at | Putative regulatory protein (DeoR family)/GI=225875440 |
| Lm4b_00496_at | Hypothetical protein of unknown function/GI=225875561 |
| Lm4b_00497_at | Conserved hypothetical proteins/GI=225875562 |
| Lm4b_00497_s_at | Conserved hypothetical proteins/GI=225875562 |
| Lm4b_00498_at | Putative secreted protein/GI=225875563 |
| Lm4b_00543_at | Putative HsdR type IC restriction subunit/GI=225875608 |
| Lm4b_00544_at | Putative HsdM type IC modification subunit/GI=225875609 |
| Lm4b_00545_at | Putative specificity determinant HsdS/GI=225875610 |
| Lm4b_00546_at | Putative bacteriophage integrase/GI=225875611 |
| Lm4b_00547_at | Putative specificity determinant HsdS/GI=225875612 |
| Lm4b_01443_s_at | Putative glutathione reductase/GI=225876496 |
| Lm4b_01608_at | Hypothetical protein of unknown function/GI=225876660 |
| Lm4b_02561_s_at | Hypothetical protein of unknown function/GI=225877602 |
| Lm4b_02686_s_at | Putative gluconate kinase/GI=225877725 |
| Lm4b_02690_s_at | cydC Putative ABC transporter required for expression of cytochrome BD/GI=225877729 |
| Lm4b_02795_x_at | Hypothetical protein of unknown function/GI=225877834 |
| LMBG_00762_s_at | predicted protein |
| LMBG_00763_x_at | HD domaincontaining protein/Pfam=PF01966.14 |
| LMBG_00856_at | conserved hypothetical protein |
| LMBG_01285_s_at | diphosphomevalonate decarboxylase/Pfam=PF08544.5 |
| LMBG_02369_x_at | inorganic pyrophosphatase |
| LMBG_02370_x_at | conserved hypothetical protein |
| LMBG_02371_s_at | predicted protein |
| LMBG_02373_at | replicationassociated protein RepB |
| LMBG_02373_s_at | replicationassociated protein RepB |
| LMBG_02374_s_at | conserved hypothetical protein |
| LMBG_02375_at | oxidoreductase |
| LMBG_02376_at | predicted protein/Pfam=PF00440.15 |
| LMBG_02376_s_at | predicted protein/Pfam=PF00440.15 |
| LMBG_02377_at | predicted protein |
| LMBG_02378_at | predicted protein |
| LMBG_02378_s_at | predicted protein |
| LMBG_02379_at | conserved hypothetical protein |
| LMBG_02380_s_at | conserved hypothetical protein |
| LMBG_02522_s_at | RNA methyltransferase/Pfam=PF01938.12 |
| LMBG_03007_x_at | lipoprotein |
| LMBG_03011_x_at | lipoprotein |
| LMFG_00259_x_at | phosphotriesterase/Pfam=PF02126.10 |
| LMFG_01112_x_at | conserved hypothetical protein/Pfam=PF00923.11 |
| LMFG_01521_x_at | conserved hypothetical protein |
| LMFG_01791_at | conserved hypothetical protein |
| LMFG_01791_x_at | conserved hypothetical protein |
| LMFG_02291_x_at | MutTnudix family protein/Pfam=PF00293.20 |
| LMFG_03051_x_at | inorganic pyrophosphatase |
| LMFG_03054_s_at | predicted protein |
| LMFG_03054_x_at | predicted protein |
| LMFG_03185_x_at | phage transcriptional activator |
| LMHCC_0206_at | pyridine nucleotide-disulfide oxidoreductase family protein/GI=217332774 |
| LMHCC_0632_at | prephenate dehydrogenase/GI=217333194 |
| LMHCC_0752_s_at | conserved hypothetical protein/GI=217333312 |
| LMHCC_1156_s_at | conserved hypothetical protein/GI=217333710 |
| LMHCC_1331_x_at | phosphoglycerate mutase family protein/GI=217333882 |
| LMHCC_1560_s_at | hypothetical protein/GI=217334110 |
| LMHCC_1903_x_at | fibronectin-binding protein/GI=217334450 |
| LMHCC_1914_s_at | flgC flagellar basal-body rod protein FlgC/GI=217334461 |
| LMHCC_2150_x_at | MutTnudix family protein/GI=217334694 |
| LMHCC_2165_x_at | cell wall surface anchor family protein/GI=217334709 |
| LMHCC_2294_s_at | inorganic pyrophosphatase/GI=217334838 |
| LMHCC_2294_x_at | inorganic pyrophosphatase/GI=217334838 |
| LMHCC_2315_x_at | thiE thiamine-phosphate pyrophosphorylase/GI=217334858 |
| LMHCC_2489_s_at | conserved hypothetical protein/GI=217335030 |
| LMHCC_2600_x_at | glyoxylate reductase (Glycolate reductase)/GI=217335141 |
| LMHCC_2705_s_at | major facilitator family transporter/GI=217335246 |
| LMHCC_2836_x_at | phosphosugar-binding transcriptional regulator, RpiR family/GI=217335374 |
| LMHG_01678_x_at | Disomer specific 2hydroxyacid dehydrogenase/Pfam=PF02826.11 |
| LMHG_01732_x_at | polysaccharide biosynthesis family protein/Pfam=PF01943.9 |
| LMHG_01915_x_at | conserved hypothetical protein/Pfam=PF00440.15 |
| LMHG_01990_x_at | PTS system protein/Pfam=PF02378.10 |
| LMHG_03318_s_at | FtsKSpoIIIE family protein |
| LMIG_02842_x_at | cell wall surface anchor family protein/Pfam=PF00746.13 |
| LMJG_00033_s_at | conserved hypothetical protein |
| LMJG_00034_s_at | conserved hypothetical protein |
| LMJG_03011_x_at | predicted protein |
| LMKG_01794_x_at | predicted protein |
| LMKG_02504_x_at | ATP synthase F1/Pfam=PF02874.15 |
| LMLG_00089_x_at | predicted protein |
| lmo0078_x_at | GI=16409437 |
| lmo0479_s_at | putative secreted protein/GI=16409855 |
| lmo1092_at | GI=16410494 |
| lmo1556_s_at | hemC GI=16410985 |
| lmo1597_s_at | GI=16411026 |
| lmo2749_at | GI=16412249 |
| LMOf2365_0124_s_at | ROK family protein/GI=46879610 |
| LMOf2365_0323_at | hypothetical protein/GI=46879809 |
| LMOf2365_0323_x_at | hypothetical protein/GI=46879809 |
| LMOf2365_0325_at | putative type II restriction enzyme Sau3AI/GI=46879810 |
| LMOf2365_0326_at | DNA-binding protein/GI=46879811 |
| LMOf2365_0326_s_at | DNA-binding protein/GI=46879811 |
| LMOf2365_0327_at | C-5 cytosine-specific DNA methylase family protein/GI=46879812 |
| LMOf2365_0327_s_at | C-5 cytosine-specific DNA methylase family protein/GI=46879812 |
| LMOf2365_0328_s_at | conserved domain protein/GI=46879813 |
| LMOf2365_0383_at | conserved hypothetical protein/GI=46879867 |
| LMOf2365_0498_x_at | cell wall surface anchor family protein/GI=46879980 |
| LMOf2365_0504_s_at | HD domain protein/GI=46879986 |
| LMOf2365_0674_s_at | putative transaldolase/GI=46880155 |
| LMOf2365_0826_s_at | spermidineputrescine ABC transporter, spermidineputrescine-binding protein/GI=46880306 |
| LMOf2365_0977_at | nagB glucosamine-6-phosphate isomerase/GI=46880456 |
| LMOf2365_1066_s_at | moaD molybdenum cofactor biosynthesis protein D/GI=46880544 |
| LMOf2365_1138_s_at | ABC transporter, ATP-bindingpermease protein/GI=46880615 |
| LMOf2365_1275_at | hydrolase, alphabeta fold family/GI=46880752 |
| LMOf2365_1421_s_at | conserved hypothetical protein/GI=46880898 |
| LMOf2365_1465_x_at | zurM2 zinc ABC transporter, permease protein/GI=46880942 |
| LMOf2365_1747_s_at | putative membrane protein/GI=46881223 |
| LMOf2365_1748_at | ABC transporter, ATP-binding protein/GI=46881224 |
| LMOf2365_1748_x_at | ABC transporter, ATP-binding protein/GI=46881224 |
| LMOf2365_1749_at | transcriptional regulator, GntR family/GI=46881225 |
| LMOf2365_1749_s_at | transcriptional regulator, GntR family/GI=46881225 |
| LMOf2365_1818_x_at | conserved hypothetical protein/GI=46881293 |
| LMOf2365_1947_at | conserved hypothetical protein TIGR00275/GI=46881421 |
| LMOf2365_2238_s_at | gpm phosphoglycerate mutase/GI=46881710 |
| LMOf2365_2416_at | leucine rich repeat domain protein/GI=46881888 |
| LMOf2365_2542_s_at | bacterial extracellular solute-binding protein, family 5/GI=46882013 |
| LMOf2365_2544_s_at | putative amidase/GI=46882015 |
| LMOf2365_2567_at | hypothetical protein/GI=46882039 |
| LMOf2365_2567_x_at | hypothetical protein/GI=46882039 |
| LMOf2365_2568_s_at | putative lipoprotein/GI=46882040 |
| LMOf2365_2620_x_at | phosphotriesterase family protein/GI=46882092 |
| LMOf2365_2626_s_at | hypothetical protein/GI=46882098 |
| LMOf2365_2627_at | ABC transporter, ATP-binding protein/GI=46882099 |
| LMOf2365_2628_at | putative membrane protein/GI=46882100 |
| LMOf2365_2629_s_at | putative membrane protein/GI=46882101 |
| LMOf2365_2630_s_at | putative membrane protein/GI=46882102 |
| LMOf2365_2631_s_at | conserved hypothetical protein/GI=46882103 |
| LMOf2365_2649_x_at | putative membrane protein/GI=46882121 |
| LMOf2365_2688_at | putative PTS system, cellobiose-specific, IIC component/GI=46882160 |
| LMOf2365_2760_s_at | putative glutamate--cysteine ligaseamino acid ligase/GI=46882232 |
| LMOf2365_2794_s_at | conserved hypothetical protein/GI=46882266 |
| LMOf2365_2795_s_at | conserved hypothetical protein/GI=46882267 |
| LMOf2365_2796_s_at | conserved hypothetical protein/GI=46882268 |
| LMOf6854_0090_x_at | D-isomer specific 2-hydroxyacid dehydrogenase family protein/GI=47016493 |
| LMOf6854_0494_x_at | hypothetical protein/GI=47016827 |
| LMOf6854_0646_s_at | conserved hypothetical protein/GI=47013873 |
| LMOf6854_0786_x_at | conserved hypothetical protein/GI=47016376 |
| LMOf6854_1649_s_at | conserved hypothetical protein/GI=47013861 |
| LMOf6854_2316_s_at | amino acid ABC transporter, permease protein, HisGluGlnArgopine family/GI=47013921 |
| LMOG_01716_at | predicted protein |
| LMOh7858_0330_s_at | PRDPTS system IIA 2 domain protein/GI=47020111 |
| LMOh7858_0533_s_at | conserved domain protein/GI=47019129 |
| LMOh7858_1994_s_at | conserved hypothetical protein/GI=47018383 |
| LMPG_03076_x_at | predicted protein |
| LMRG_02134_x_at | conserved hypothetical protein |
| LMRG_02385_x_at | dipeptideoligopeptide ABC transporter/Pfam=PF00528.14 |
| LMRG_02859_x_at | predicted protein |
| LMRG_02864_at | conserved hypothetical protein |
| LMRG_02931_at | predicted protein |
| LMSG_00485_s_at | conserved hypothetical protein |
| LMSG_01908_x_at | conserved hypothetical protein/Pfam=PF00923.11 |
| LMSG_02462_x_at | oligopeptide ABC transporter/Pfam=PF00528.14 |
| LMSG_02922_x_at | predicted protein |
| LMSG_02960_s_at | conserved hypothetical protein |
| LMSG_03151_x_at | predicted protein |

NK: Not known
